# Advanced 3D-Printed Flexible Composite Electrodes of Diamond, Carbon Nanotubes, and Thermoplastic Polyurethane

**DOI:** 10.1021/acsapm.4c02748

**Published:** 2024-11-19

**Authors:** Simona Baluchová, Stach van Leeuwen, Baris Kumru, Josephus G. Buijnsters

**Affiliations:** † Department of Precision and Microsystems Engineering, Faculty of Mechanical Engineering, Delft University of Technology, Mekelweg 2, 2628 CD Delft, The Netherlands; ‡ Department of Analytical Chemistry, Faculty of Science, Charles University, Albertov 6, 128 00 Prague, Czech Republic; § Department of Aerospace Structures and Materials, Faculty of Aerospace Engineering, Delft University of Technology, Kluyverweg 1, 2629 HS Delft, The Netherlands

**Keywords:** 3D printing, thermoplastic polyurethane, boron-doped diamond microparticles, multi-walled carbon nanotubes, flexible composite electrodes, electrochemical characterization

## Abstract

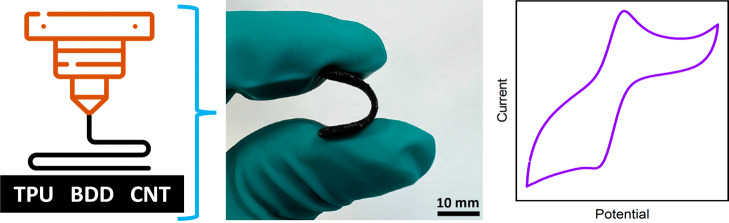

In this work, we pioneered the preparation of diamond-containing flexible electrodes using 3D printing technology. The herein developed procedure involves a unique integration of boron-doped diamond (BDD) microparticles and multi-walled carbon nanotubes (CNTs) within a flexible polymer, thermoplastic polyurethane (TPU). Initially, the process for the preparation of homogeneous filaments with optimal printability was addressed, leading to the development of two TPU/CNT/BDD composite electrodes with different CNT:BDD weight ratios (1:1 and 1:2), which were benchmarked against a TPU/CNT electrode. Scanning electron microscopy revealed a uniform distribution of conductive fillers within the composite materials with no signs of clustering or aggregation. Notably, increasing the proportion of BDD particles led to a 10-fold improvement in conductivity, from 0.12 S m^–1^ for TPU/CNT to 1.2 S m^–1^ for TPU/CNT/BDD (1:2). Cyclic voltammetry of the inorganic redox markers, [Ru(NH_3_)_6_]^3+/2+^ and [Fe(CN)_6_]^3–/4–^, also revealed a reduction in peak-to-peak separation (Δ*E*_p_) with a higher BDD content, indicating enhanced electron transfer kinetics. This was further confirmed by the highest apparent heterogeneous electron transfer rate constants (*k*^0^_app_) of 1 × 10^–3^ cm s^–1^ obtained for both markers for the TPU/CNT/BDD (1:2) electrode. Additionally, the functionality of the flexible TPU/CNT/BDD electrodes was successfully validated by the electrochemical detection of dopamine, a complex organic molecule, at millimolar concentrations by using differential pulse voltammetry. This proof-of-concept may accelerate development of highly desirable diamond-based flexible devices with customizable geometries and dimensions and pave the way for various applications where flexibility is mandated, such as neuroscience, biomedical fields, health, and food monitoring.

## 1 Introduction

Diamond, known for its exceptional mechanical hardness, chemical inertness, wide band gap (5.5 eV), high electrical resistivity (exceeding 10^16^ Ω cm) and thermal conductivity (2000 W m^–1^ K^–1^), has been widely used in diverse fields such as thermal management, optical systems, electronics, biomedical devices, and quantum technology.^1,2^ The electrical properties of diamond are adjustable through boron doping to range from insulating to (semi-)conductive, making it ideal for electrochemistry and (bio)sensor applications.^3,4^ Traditionally, (boron-doped) diamond is synthesized as a thin film on rigid substrates (e.g., silicon, tungsten) using chemical vapor deposition (CVD) at temperatures above 700 °C.^1,3^ However, as applications advance, more complex designs, often integrated with flexible platforms, are demanded. In general, flexible, polymer-based electrodes are highly desirable in the (bio)electronic^5−7^ and biomedical fields for applications such as neural implants, which stimulate or record neuron activity,^8^ skin wearable electronics^9^ and sensors for non-invasive clinical analysis in sweat,^10,11^ tissue and organ engineering,^12^ and soft robotics.^13^ The high synthesis temperatures applied in CVD prevent the direct deposition of diamond onto flexible polymeric substrates. Although a few attempts have been made to transfer thin-film CVD diamond to flexible supports,^14−16^ these processes are typically multi-step, involve complex procedures (e.g., photolithography, etching), and are laborious and costly, which naturally impedes the production and practical application of flexible diamond-based sensors.

An alternative approach to circumvent these challenges could involve the use of diamond in particulate form rather than as a CVD thin film, potentially combined with an appropriate printing technique. Printing is generally regarded as a straightforward, cost-effective method of manufacturing, which offers design flexibility, rapid prototyping, and the capability of producing custom sensing devices with specific geometries and sizes while minimizing waste. Although nanomaterials are widely recognized as excellent components for functional filaments in technologies such as 3D printing,^17,18^ (boron-doped) diamond particles have not yet been reported for the fabrication of flexible electrodes.

Recent studies have explored the use of diamond microparticles in 3D printing, although the literature on this topic remains limited. Paull and co-workers pioneered the development of thermally conducting composite materials for 3D printing, incorporating micron-sized diamond powders.^19,20^ For example, by using stereolithography or fused-deposition modeling, diamond microparticles were embedded in either an acrylate resin^19^ or a thermoplastic acrylonitrile butadiene styrene polymer.^20^ The resulting composite materials were utilized to develop 3D-printed prototypes, such as heat sinks and cooling coils, for thermal management applications. Additionally, a 3D printable composite comprising 60 wt % boron-doped diamond (BDD) microparticles (2–3 μm), 2 wt % LiCl, and acrylonitrile butadiene styrene was developed and applied to fabricate humidity sensors using a low-cost fused deposition modeling 3D printer.^21^ In contrast, there were also studies incorporating either micro- or nano-sized diamond particles with inkjet printing^22,23^ or screen printing.^24−26^ None of these works has yet explored the integration of diamond particles with flexible substrates, indicating an unexplored research field. Typically, poly-lactic acid has been the polymer of choice for fabricating bespoke conductive filaments, commonly using carbon black and graphite as fillers,^18^ and for subsequent use in 3D printing electrochemical sensors. However, there is considerable potential for developing conductive filaments using alternative polymers that offer distinctive properties, such as the flexibility provided by thermoplastic polyurethane (TPU).

In this work, we propose an innovative approach for the preparation of diamond-containing flexible electrodes using a 3D printing technique, particularly fused deposition modeling. The key components were commercially available BDD microparticles and multi-walled carbon nanotubes (CNTs), which were uniquely combined for the first time and embedded within a biocompatible flexible TPU. Particularly, we fabricated two TPU/CNT/BDD composite electrodes with CNT:BDD – filler weight ratio of 1:1 and 1:2, and compared them to a TPU/CNT electrode to assess the impact of BDD incorporation. The morphology and electrochemical characteristics were thoroughly investigated and evaluated using scanning electron microscopy and voltammetry, respectively. To our knowledge, this is the first report on the fabrication of a 3D-printed, flexible, and diamond-containing electrode material intended for electrochemical applications.

## 2 Experimental Section

Information on chemicals and materials used together with full experimental details on the fabrication of the flexible electrodes and their electrical characterization is presented in Supporting Information (SI).

### 2.1 Composite Pellets Preparation

**Figure 1:**
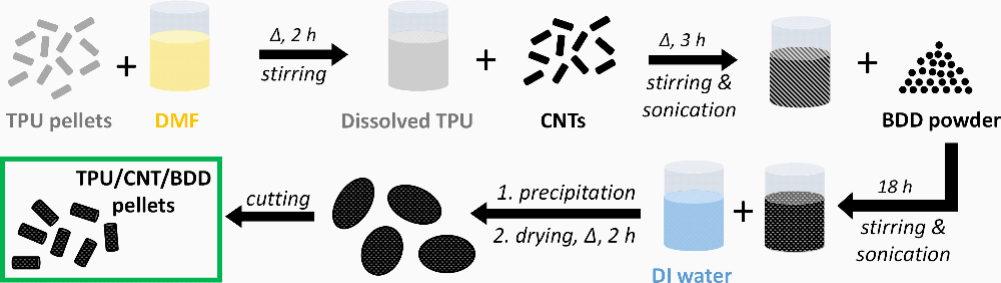
Schematic illustration of the fabrication of TPU/CNT/BDD composite pellets.

A schematic overview of the fabrication of TPU/CNT/BDD composite pellets is shown in Figure 1. Briefly, in the first stage, the fabrication process consisted of several steps involving dissolution, heating, stirring, and ultrasonication to obtain a homogeneous TPU/CNT/BDD (in dimethylformamide, DMF) mixture. The dispersion was then precipitated in deionized water to obtain the composite precipitates. The resulting precipitates were first dried and then manually cut into 10–20 mm pellets, which were used for filament extrusion.

### 2.2 Composite Filament Extrusion

**Figure 2:**
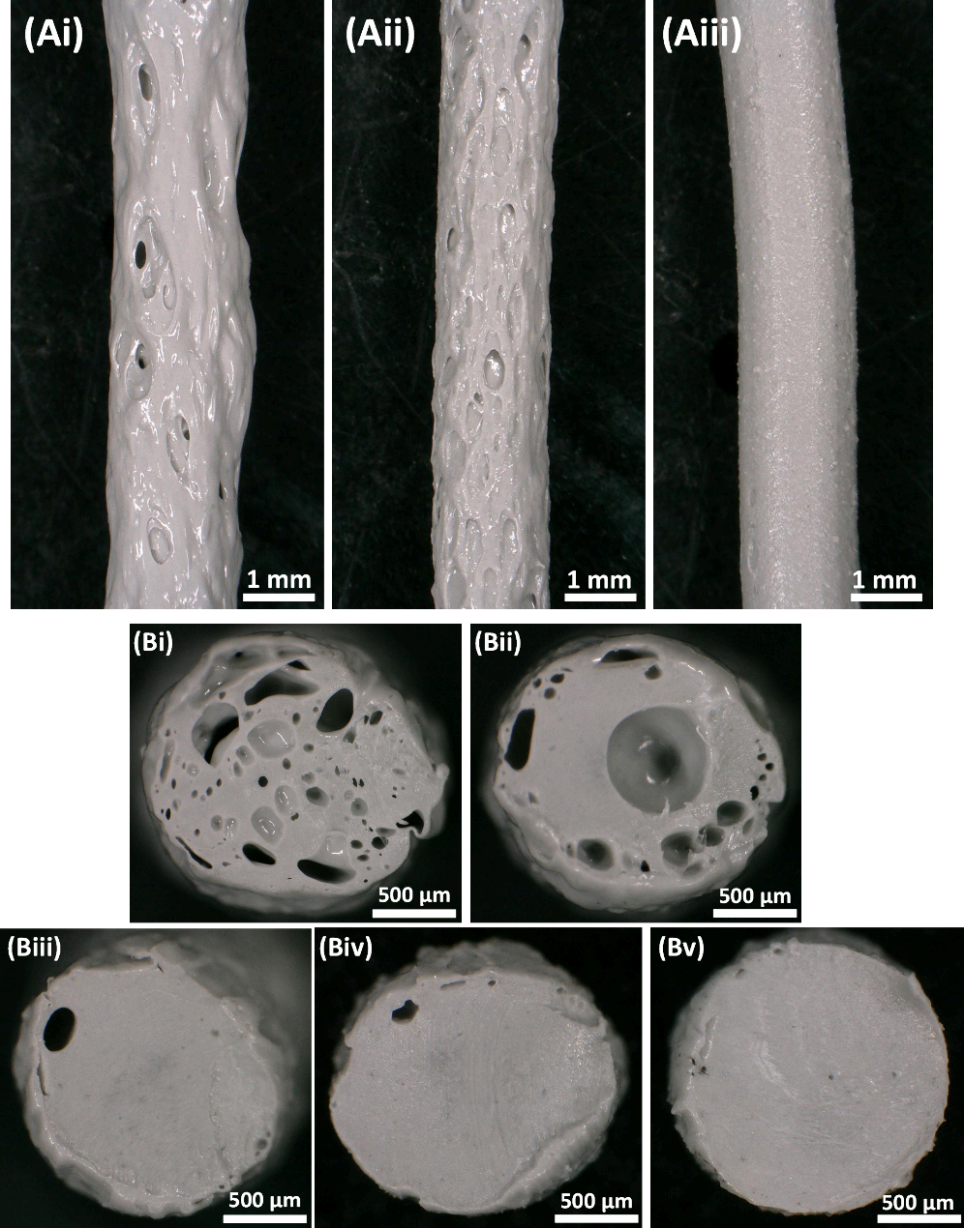
Optical micrographs showing the effect of the total number of consecutive extrusions on: (A) the filament surface roughness after (i) 1, (ii) 3, and (iii) 5 extrusion steps; (B) the corresponding composite filament cross-section with (i)–(v) representing 1 to 5 extrusion steps, respectively.

The manufactured pellets were fed to the hopper element of the extruder (Felfil, Italy) and extruded via a 1.75 mm nozzle. The extrusion was performed using temperatures ranging from 195 to 215 °C, extrusion speed of 3–5 rpm, and pulling speed of 0.4–0.6 m min^–1^. Filaments were re-extruded multiple times until a dense, non-porous structure was achieved (see Figure 2), rendering them suitable for 3D printing. A modified Prusa i3MK3S+ fused deposition modeling printer (Prusa Research, Czech Republic), adapted to withstand abrasiveness of diamond, was utilized to fabricate standalone 3D-printed flexible electrodes of 25 mm width × 25 mm length × 1 mm thickness, using printing parameters summarized in Table S1, with an average printing duration of 3 min per sample.

### 2.3 Characterization Techniques

The particle size distribution of the BDD powder, dispersed in deionized water, was measured using a Mastersizer 3000 (Malvern Instruments, Worcestershire, UK), equipped with a Hydro SM sample dispersion unit through laser light scattering. The BDD powder, filament cross-section, and surface morphology were visualized using a JEOL-JSM6010LA scanning electron microscope in secondary electron imaging mode, operating at an acceleration voltage of 5 kV. Before imaging, the samples were coated with a thin gold layer using a JEOL JFC-1300 auto fine coater to improve conductivity. Optical images of the extruded filaments and 3D-printed electrodes were captured by using a digital microscope (VHX-6000, Keyence, Belgium).

Electrochemical measurements, including cyclic voltammetry (CV) and differential pulse voltammetry (DPV), were conducted at a laboratory temperature of 23 °C using an Autolab PGSTAT 128N, operated by Nova 2.1 software (Metrohm, The Netherlands). The experimental setup consisted of a conventional three-electrode system with the 3D-printed composite electrodes serving as the working electrodes (WEs), a silver–silver chloride (3 mol L^–1^ NaCl) reference electrode (RE-1BP from ALS, Japan), and a 25 cm long platinum wire counter electrode (redox.me, Sweden). The WE was secured in a 25 × 25 mm^2^ sample holder with a 1 cm^2^ aperture (redox.me, Sweden) and submerged in the measuring solution within a glass beaker. Besides, the CV technique was utilized to assess double-layer capacitance (*C*_dl_), using scans recorded in a 0.5 mol L^–1^ KNO_3_ solution over the potential range from −0.5 V to +0.5 V. *C*_dl_ was evaluated using eq 1:
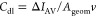
1where Δ*I*_AV_ represents the average background current difference between the forward (anodic) and reversed (cathodic) scan at a potential of 0 V, *A*_geom_ denotes the geometric surface area (1 cm^2^), and *v* signifies the scan rate (100 mV s^–1^).

The Nicholson method^27^ was applied to estimate the apparent heterogeneous electron transfer (HET) rate constant (*k*^0^_app_; in cm s^–1^) using eq 2:
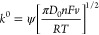
2where ψ is a dimensionless kinetic parameter obtained from the peak-to-peak separation (Δ*E*_p_) values^27^ assessed from CV measurements, *D*_0_ is the diffusion coefficient (7.6 × 10^–6^ cm^2^ s^–1^ for [Fe(CN)_6_]^3–/4–^ ^28^ and 5.5 × 10^–6^ cm^2^ s^–1^ for [Ru(NH_3_)_6_]^3+/2+^ ^29^), *n* is the number of electrons (1), *F* is the Faraday constant (96,485 C mol^–1^), *v* is the scan rate (10 mV s^–1^), *R* is the gas constant (8.314 J K^–1^ mol^–1^), *T* is the temperature (298 K), and π is 3.14.

DPV measurements were conducted in a solution of dopamine in phosphate buffered saline (PBS, 10 mmol L^–1^, pH 7.4), using a pulse amplitude of +25 mV, a pulse width of 50 ms, a potential step of 2.5 mV, and a scan rate of 5 mV s^–1^.

## 3 Results and Discussion

### 3.1 Composite Filament Fabrication

Prior to filament fabrication, commercially acquired BDD powder was subjected to scanning electron microscopy and particle size analysis (see Figure S1). The scanning electron micrograph of the powder indicates that the majority of the particles possess a blocky and irregular shape, and are submicrometer in size. Results of laser diffraction analysis confirmed that the BDD particle sizes range from 0.1 to 1.0 μm, with the most prevalent sizes being approximately 0.2–0.4 μm.

Initially, we designed and subsequently converted three distinct electrode composite formulations, namely, TPU/CNT, TPU/CNT/BDD (1:1), and TPU/CNT/BDD (1:2), into printable filament materials. To ensure high electrical conductivity and to reach the percolation threshold, we formulated our compositions with 10 wt % CNTs. In our work, CNTs were used as a primary filler owing to (i) their highly advantageous properties, such as high electrical conductivity, good processability, and excellent recyclability in thermoplastics, and (ii) their suitable dimensions, particularly the average length of 1.5 μm. The longer CNTs are especially beneficial, as they more easily form an intertwined structure within the polymer matrix (compared to spherical nanofillers like carbon black), helping to reach the percolation threshold more effectively. The amount of added BDD microparticles, serving as the secondary filler, was varied to further optimize the electrochemical performance of the 3D-printed electrodes and to investigate the effects of BDD incorporation. In addition, a filament composed solely of TPU was prepared and used for comparison purposes.

Figure S2(A) provides an overview of all extruded filaments in their final form. Meanwhile, to achieve such optimized filaments suitable for 3D printing, a number of extrusion parameters must be carefully addressed. As can be seen in Figure 2(Ai), the single-extruded filaments exhibited significant rupture and porosity with inconsistent diameters, which was attributed to precipitation in water involved in the manufacturing method of the composite pellets (Section 2.1). This process resulted in the formation of tiny water bubbles within the pellets, leading to a porous nature after drying.

Subsequent extrusions progressively improved the filament quality (Figure 2(Aii)). Notably, by the fifth extrusion, the filament was dense and homogeneous with negligible porosity and, most importantly, consistent diameter lengthwise (see Figure 2(Aiii)). Optical micrographs of the filament cross sections after five sequential extrusions, depicted in Figure 2(Bi)–2(Bv), showed reduced porosity with each extrusion. These results confirmed the observation reported for Figure 2(A) above. Our results are in agreement with those reported by Waheed et al.^20^ in that a minimum of five to six extrusions was necessary to achieve dense, non-porous filaments. Their results on microdiamond acrylonitrile butadiene styrene composites also demonstrated a gradual enhancement in homogeneity and reduction in voids through consecutive extrusions. However, the potential degradation of filament material due to repeated high-temperature exposure must also be considered. Cieslik et al.^30^ proved that repeated extrusion cycles degrade the polymer matrix of a conductive poly-lactic acid filament, leading to diminished electrical conductivity from 7 S m^–1^ to 4 S m^–1^ and deteriorated electrochemical properties, which was attributed to the formation of conductive aggregates during re-extrusion.

The volume resistivity and electrical conductivity of the 3D-printed composites (see Figure S2) was determined using Equations S1 and S2, as described in Section A5 of the SI. After introducing BDD particles in the filament composite, the volume resistivity gradually dropped with increasing BDD concentration and a value of 92 Ω cm was recorded for the TPU/CNT/BDD (1:2) filament. This corresponds to a conductivity of 1.2 S m^–1^, which is one order of magnitude higher than the value measured for the TPU/CNT filament.

In addition, we successfully manufactured TPU filaments containing only BDD microparticles at concentrations of 10, 20, and even 40 wt %. These filaments were utilized to 3D print ’electrodes’. However, they all lacked conductivity, indicating that the current doping level of BDD particles (specified as 710 ppm by the manufacturer) was insufficient for standalone conductivity. Nevertheless, when BDD microparticles were combined with CNTs, they significantly contributed to conductivity. This synergistic effect is evidenced by the clear differences in conductivity (Figure S2(B)) and electrochemical performance (see Section 3.3 below) between TPU/CNT and TPU/CNT/BDD electrodes.

Unlike most studies that focused on the use of a single type of conductive particle, our research explores the synergy of a binary conductive particle system. The concept of utilizing two different types of fillers has been previously proposed.^31^ Particularly in the case of hybrid CNT composites, the incorporation of the micro-scale secondary fillers, such as BDD microparticles, generates an excluded volume that leads to a denser CNT network. This in turn increases the number of effective conducting paths and enhances the electrical conductivity of the composite. Our observations thus align with previously reported modeling,^31^ which demonstrated that adding micro-sized silica particles to a fixed CNT content reduces the total resistance (and increases the conductivity) of the CNT/silica composite by increasing the number of current paths. These findings further support the benefits of a binary particle approach in enhancing the electrical conductivity of the composite.

### 3.2 Morphological Characterization

**Figure 3:**
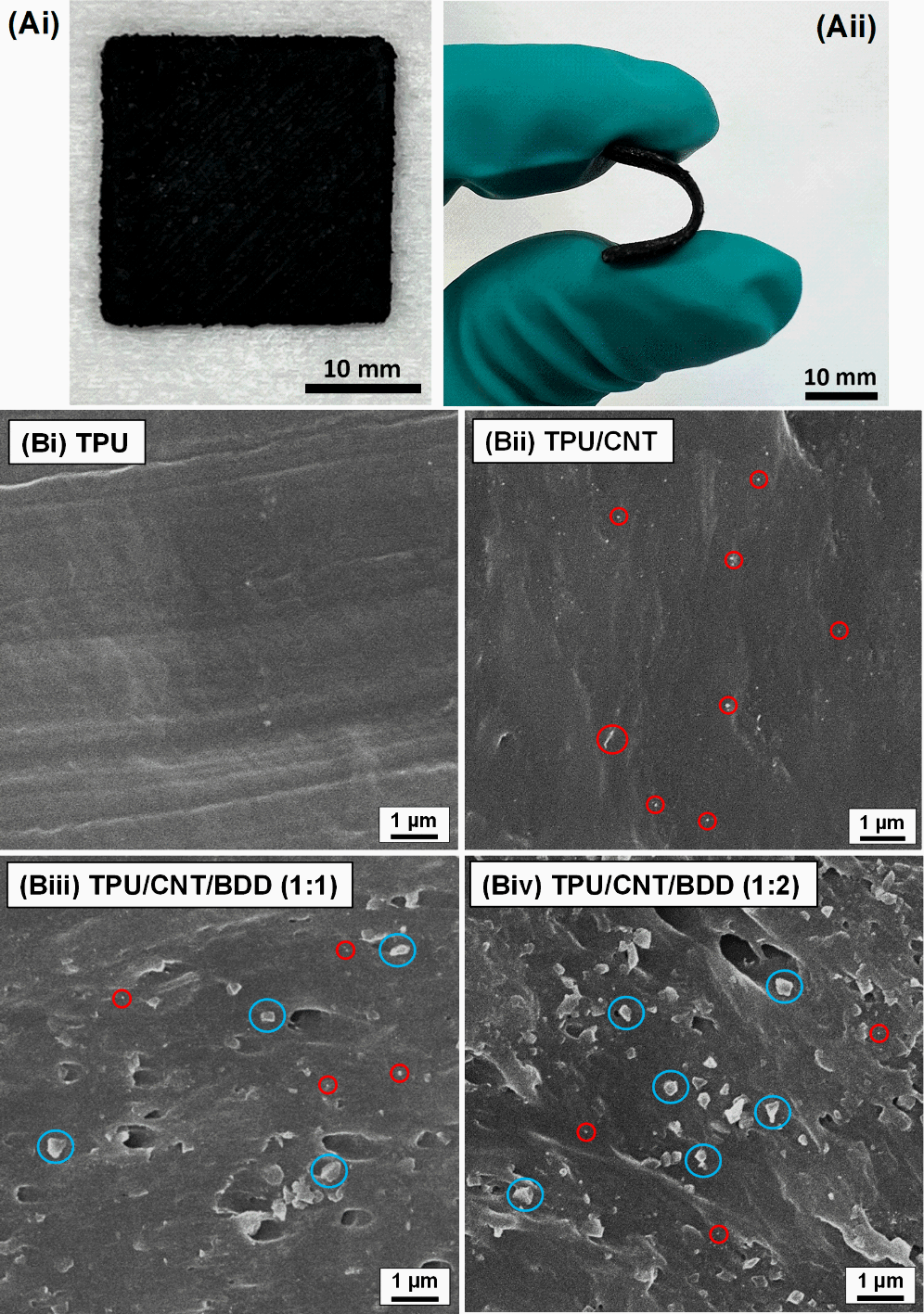
(A) Optical images displaying (Ai) the 3D-printed composite electrode and (Aii) its flexibility. (B) Scanning electron micrographs recorded at the cross-section areas of different 3D-manufactured (i) TPU and (ii-iv) TPU/filler-based composites. Red and blue circles highlight several selected CNT and BDD particles, respectively.

Next, the composite filaments were used to 3D print several standard electrode designs (e.g., square disks); an example is shown in Figure 3(Ai). The flexibility of these electrodes is clearly demonstrated in Figure 3(Aii) and Video S1 (available in the SI). Scanning electron microscopy was employed to analyze the cross-section morphology of 3D-printed neat TPU and three composite materials, and the micrographs obtained are depicted in Figure 3(B). The scanning electron micrograph of neat TPU, shown in Figure 3(Bi), reveals a smooth homogeneous morphology. For the TPU/CNT composite (Figure 3(Bii)), a similar degree of smoothness is observed together with the presence of tiny white spots (circled in red). These bright spots are often affiliated with conductive particles,^32,33^ and they are thus ascribed to the presence of CNT fillers. The absence of significantly clustered or aggregated CNTs, evidenced by the bright spot diameters not exceeding 100 nm and corresponding to roughly ≤10 CNTs, indicates a homogeneous dispersion within the composite. Notable morphological changes became evident after the introduction of BDD particles. The cross-sections exhibited increasing roughness correlating with higher BDD concentrations, as shown in Figure 3(Biii) and 3(Biv). This roughness was accompanied by a visible increase in the porosity. Also, more BDD particles, highlighted by blue circles, can be identified in the scanning electron micrographs with rising filler concentrations, and importantly, their distribution remains relatively uniform.

### 3.3 Electrochemical Characterization

All three 3D-printed composite electrodes were electrochemically characterized using CV before and after a surface treatment procedure involving a KNO_3_ supporting electrolyte and two respective redox markers, [Ru(NH_3_)_6_]^3+/2+^ and [Fe(CN)_6_]^3–/4^^–^, as described in Section 3.3.1. The measurements in KNO_3_ provided valuable information on *C*_dl_ values of both treated and untreated flexible electrodes, while experiments with [Ru(NH_3_)_6_]^3+/2+^ and [Fe(CN)_6_]^3–/4–^ were used in optimizing the treatment procedure as well as in studying the HET kinetics of the treated composite electrodes. [Ru(NH_3_)_6_]^3+/2+^ is a well-known outer-sphere redox marker, whose redox reaction is controlled by simple diffusion and is less impacted by surface characteristics.^23,34^ In contrast, [Fe(CN)_6_]^3–/4–^ is a well-characterized inner-sphere redox marker involving specific surface interactions,^23,34^ making it sensitive to the electrode surface properties.

#### 3.3.1 Post-Printing Electrode Treatment

**Figure 4:**
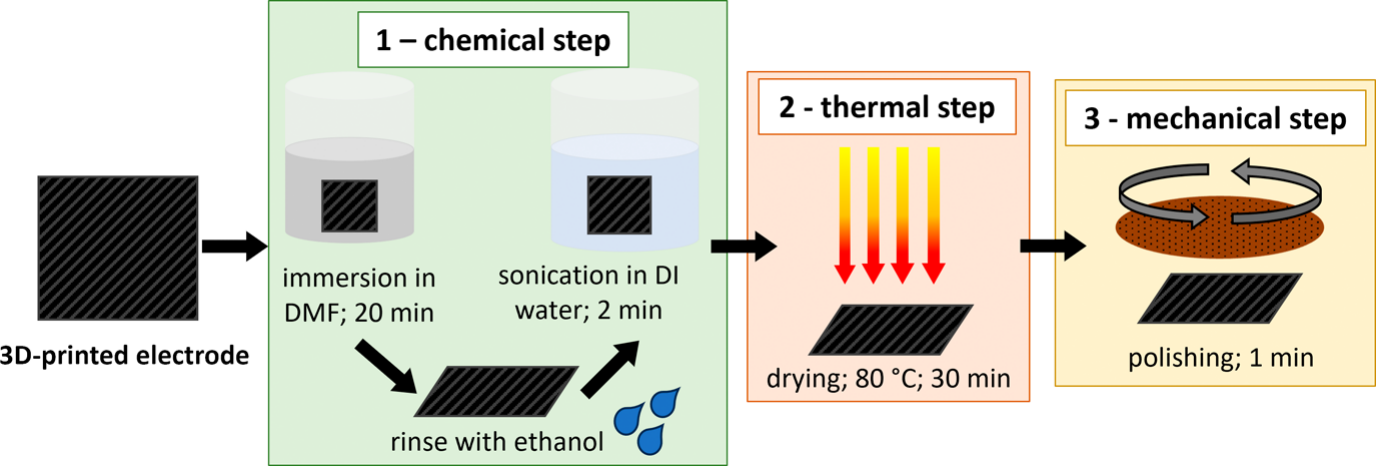
Schematic illustration of the complete treatment procedure for the surface of the 3D-printed composite electrodes.

Very often, 3D-printed composite electrodes require surface treatments, because the conductive material is encapsulated by the (thermoplastic) polymer. Consequently, surface treatment strategies must be employed to mitigate this issue by partially removing the insulating polymer layer from the electrode surface. Such strategies include more traditional approaches, such as chemical^35−37^ and electrochemical activation,^38−40^ mechanical polishing,^36,38^ and thermal annealing,^41^ as well as a recently reported reagent-free approach of spark-discharge activation.^42^ Notably, existing literature predominantly focuses on the use of poly-lactic acid as the main polymer in composite electrodes subjected to sensing applications. Among the most efficient methods are saponification of poly-lactic acid in sodium hydroxide,^40^ and partial dissolution in chlorinated organic solvents^37^ or DMF.^35^ These studies inspired the development of a surface activation treatment for TPU-based composites, as there have been no previous reports of TPU being employed as the primary polymer in 3D-printed electrodes specifically for electrochemical detection. The adopted electrode surface treatment procedure for this study is schematically illustrated in Figure 4, which depicts three main steps. In the first step, the as-printed electrodes were placed in DMF solvent and subsequently rinsed with ethanol to eliminate residual DMF. The effect of a series of incubation durations extending from 2.5 to 20 min was explored. The most significant effects, including improved peak shapes and the highest peak current intensity for redox markers, were observed after 20 min. As a result, this was selected as the optimal immersion time. Then, the electrodes underwent a 2 min sonication in deionized water, followed by the second step consisting of a 30 min drying phase in an oven set at 80 °C to remove excess water. The final, third step involved brief polishing (∼1 min) using a fine-grained sandpaper to smoothen the electrode surface. From previous studies involving both mechanical and solvent activation of poly-lactic acid–based materials,^40^ we concluded that mechanical polishing alone would not be sufficient to remove the TPU and expose the fillers. Naturally, the complete three-step treatment resulted in a reduction in size of the 3D-printed electrode (by ∼25%) and consequently in a minor loss of both fillers embedded in the dissolved TPU. Despite these changes, the treated electrodes retained a high degree of flexibility and mechanical integrity. No significant differences in the overall surface morphology, including the average surface roughness, were observed. The applied treatment did not notably roughen the already characteristically rough 3D-printed electrode material.

#### 3.3.2 Cyclic Voltammetry in a Supporting Electrolyte

Prior to surface treatment, the as-printed electrodes were first characterized in a 0.5 mol L^–1^ KNO_3_ supporting electrolyte, with their CV responses depicted in Figure S3(A). By estimating *C*_dl_ from the voltammograms in Figure S3(A) using eq 1, there is 3–4 times higher *C*_dl_ at BDD-containing composite electrodes caused by an increased background current. In addition, the as-printed TPU/CNT/BDD (1:1) and TPU/CNT/BDD (1:2) electrodes provided comparable CV responses, but these become much more distinct after exposure to the treatment protocol, as shown in Figure S3(B). A notable enhancement in background currents and associated *C*_dl_ values (see Table 1) was recognized for all three composite electrodes upon surface treatment (see Section 3.3.1 and Figure 4), while the increase was much more pronounced for the BDD-integrated electrodes. This can be ascribed to the treatment process, which effectively exposes conductive particles by removing excess polymer and increases the electroactive surface area. However, there is considerable room for optimizing the post-treatment procedure, particularly in reducing the capacitive currents while still exposing the fillers at the surface and enhancing electrochemical performance. Minimizing capacitive currents is particularly important when electroanalytical and sensing applications of 3D-printed composite electrodes are envisioned.

**Table 1:** Composition and Electrochemical Characteristics of the Three Different 3D-Printed Flexible Composite Electrodes^a^

	Electrode		
	TPU/CNT	TPU/CNT/BDD (1:1)	TPU/CNT/BDD (1:2)
Wt% of a component	90/10	81.8/9.1/9.1	75/8.3/16.7
**Untreated**			
*C*_dl_ (μF cm^–2^)	6	23	18
**Treated**			
*C*_dl_ (μF cm^–2^)	376	1440	4500
Δ*E*_p_ – [Ru(NH_3_)_6_]^3+/2+^ (mV)	178	127	110
*k*^0^_app_ – [Ru(NH_3_)_6_]^3+/2+^ (cm s^–1^)	5.7 × 10^–4^	9.1 × 10^–4^	1.2 × 10^–3^
Δ*E*_p_ – [Fe(CN)_6_]^3–/4–^ (mV)	>800	190	122
*k*^0^_app_ – [Fe(CN)_6_]^3–/4–^ (cm s^–1^)	-	4.6 × 10^–4^	1.1 × 10^–3^

a Reported Δ*E*_p_ and *k*^0^_app_ values are for a scan rate of 10 mV s^–1^.

Interestingly, for the TPU/CNT electrode, both untreated and treated (see red traces in Figure S3(A, B)), a relatively smaller capacitance was observed compared to TPU/CNT/BDD samples, which is likely due to the absence of BDD particles. This suggests that not only does the DMF treatment influence the resulting *C*_dl_ values but the amount of BDD particles incorporated in the composite also plays a significant role. This effect could be possibly associated with impurities, which can be present in commercially obtained CNTs and BDD powder. For example, diamond microparticles of high-pressure high-temperature origin have previously been shown to contain up to 3 wt % of elemental impurities such as Si, W, Ta, P, Al, Mn, and S.^19,43^ Similar observations were made in previous studies where metal-based impurities embedded in 3D-printed graphene/poly-lactic acid electrodes were found to significantly enhance their overall capacitance.^44^ As we did not perform any “cleaning” procedures on the high-pressure high-temperature-produced BDD particles (nor CNTs) prior to their use in developing filament formulations, these impurities could contribute to the observed effects, especially considering that the highest background currents and associated *C*_dl_ values were, indeed, recorded on the TPU/CNT/BDD (1:2) electrode, which contains the highest amount of fillers. Additionally, the finite size and limited intrinsic electrical conductivity of the BDD fillers (with a boron content of 710 ppm) may also contribute to the observed increase in capacitive currents.

#### 3.3.3 Cyclic Voltammetry in Redox Marker Solutions

**Figure 5:**
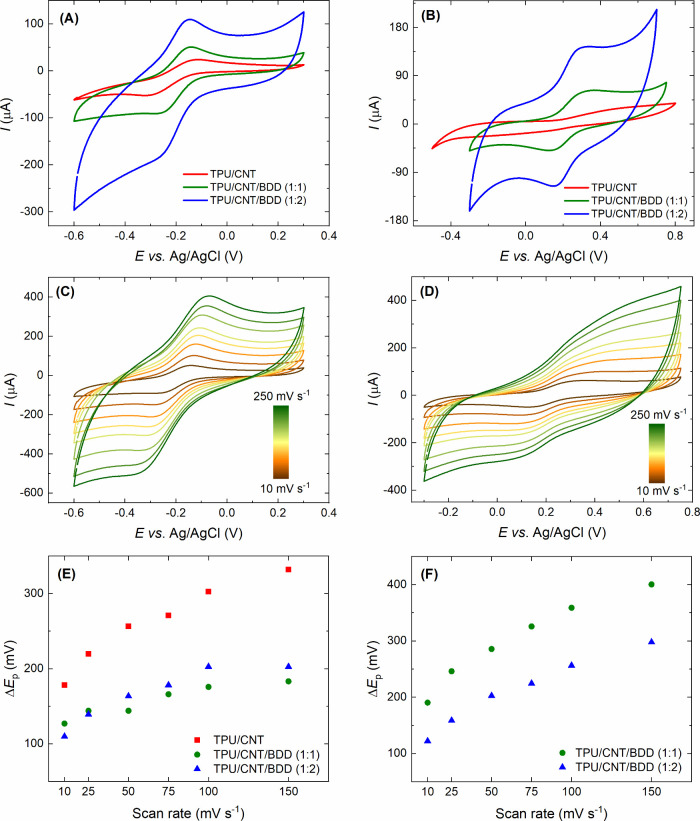
(A, B) Cyclic voltammograms recorded at a scan rate of 10 mV s^–1^ on the treated composite electrodes in solutions of (A) [Ru(NH_3_)_6_]^3+/2+^ and (B) [Fe(CN)_6_]^3–/4–^ (both 1 mmol L^–1^ in 0.5 mol L^–1^ KNO_3_). (C, D) Scan rate study performed with (C) [Ru(NH_3_)_6_]^3+/2+^ and (D) [Fe(CN)_6_]^3–/4–^ at the TPU/CNT/BDD (1:1) electrode. (E, F) Dependence of Δ*E*_p_ values on the scan rate acquired for the treated composite electrodes in solutions of (E) [Ru(NH_3_)_6_]^3+/2+^ and (F) [Fe(CN)_6_]^3–/4–^.

Next, the electrochemical properties of the composite electrodes were evaluated by cyclic voltammetry of two well-established redox markers, [Ru(NH_3_)_6_]^3+/2+^ and [Fe(CN)_6_]^3–/4–^. Initially, CVs recorded on the untreated electrodes did not display well-defined redox peaks, as shown in Figure S3(C, D). However, after the surface treatment distinct redox signals emerged, with the most intense and best-shaped peaks observed after 20 min of immersion in DMF (as also described in Section 3.3.1). The responses obtained for [Ru(NH_3_)_6_]^3+/2+^ and [Fe(CN)_6_]^3–/4–^ on the treated electrodes are displayed in Figure 5(A) and (B), respectively. Notably, despite the increase in capacitive currents following the post-treatment procedure (Figure 4), the development of distinct peaks is facilitated. From these CV measurements, peak-to-peak separation (Δ*E*_p_), was estimated (see Table 1) as an indicator of HET kinetics. As can be seen in Figure 5(A), [Ru(NH_3_)_6_]^3+/2+^ provided relatively well-defined pairs of redox peaks for all composite electrodes, while the highest Δ*E*_p_ of 178 mV, and thus the slowest HET kinetics, was recorded on the TPU/CNT electrode. In addition, this same electrode exhibited ill-defined redox peaks for the [Fe(CN)_6_]^3–/4–^ redox probe (see Figure 5(B)) with a wide Δ*E*_p_ exceeding 800 mV, suggesting sluggish HET kinetics. However, with the addition of BDD particles into the composite electrode material, the peak shape improved, and Δ*E*_p_ of both tested redox markers became smaller (see Table 1 and Figure 5(E,F)).

Further, by increasing the scan rate (*v*) from 10 to 250 mV s^–1^, cyclic voltammograms of [Ru(NH_3_)_6_]^3+/2+^ and [Fe(CN)_6_]^3–/4–^ recorded on TPU/CNT/BDD (1:1) are shown in Figure 5(C, D). Due to the lack of distinct [Fe(CN)_6_]^3–/4–^ signals on the TPU/CNT electrode, no scan rate study was conducted on this electrode type. For the other combinations of composite electrodes and redox markers, the recorded peak currents showed a linear dependence on the square root of the scan rate (Figure S4 for [Ru(NH_3_)_6_]^3+/2+^), indicating diffusion-controlled redox processes. Subsequently, based on these measurements, the relationship between Δ*E*_p_ of the redox probe and the scan rate was plotted, see Figure 5(E, F). Notably, [Ru(NH_3_)_6_]^3+/2+^ exhibited higher Δ*E*_p_ (by 50 to 150 mV on average, as depicted in Figure 5(E)) on the TPU/CNT electrode. We used these results to infer impeded HET at the TPU/CNT electrode. The two different BDD-integrated electrodes showed similar Δ*E*_p_ values for [Ru(NH_3_)_6_]^3+/2+^, but notable differences emerged with [Fe(CN)_6_]^3–/4–^, presumably due to its inner-sphere character and sensitivity to the electrode surface properties. Figure 5(F) clearly illustrates that the TPU/CNT/BDD (1:2) electrode manifested the lowest Δ*E*_p_, supporting the fastest HET kinetics, which can be accounted for by the abundance of both CNT and BDD particles and their synergistic interactions.

Next, Δ*E*_p_ values obtained from CVs at a scan rate of 10 mV s^–1^ were used to derive the ψ parameters (ψ is logarithmically related to Δ*E*_p_), which were subsequently applied in the Nicholson method to estimate *k*^0^_app_ values (see eq 2). The estimated values are summarized in Table 1 and demonstrate that for [Ru(NH_3_)_6_]^3+/2+^, *k*^0^_app_ on the composite electrodes containing BDD microfillers were 1.6–2.1 times higher than those for the TPU/CNT electrode (*k*^0^_app_ of 5.7 × 10^–4^ cm s^–1^), indicating faster HET kinetics on BDD-containing electrodes. Similarly, *k*^0^_app_ for [Fe(CN)_6_]^3–/4–^ measured on TPU/CNT/BDD samples confirmed that higher BDD content led to enhanced HET kinetics. The fastest electron transfers, with *k*^0^_app_ values on the order of 1 × 10^–3^ cm s^–1^, were observed for TPU/CNT/BDD (1:2) with both redox markers. Notably, the *k*^0^_app_ values obtained in this study align well with previously reported values for 3D-printed electrodes composed of graphene/poly-lactic acid, which also fall within the 10^–3^ to 10^–4^ cm s^–1^ range.^40,45^

### 3.4 Differential Pulse Voltammetry in Dopamine Solution

**Figure 6:**
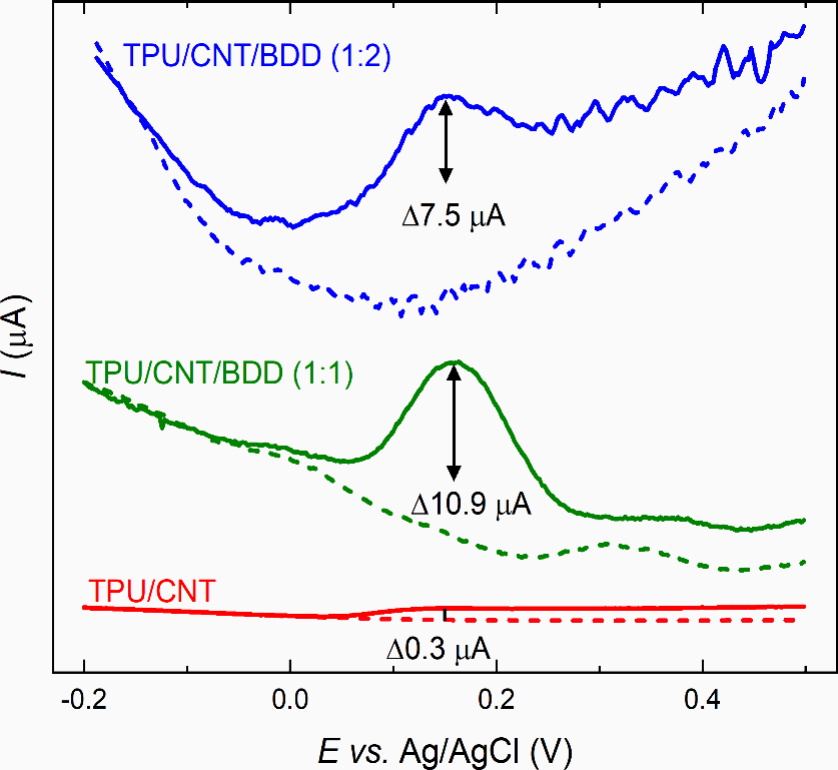
Differential pulse voltammograms of 1 mmol L^–1^ dopamine in 10 mmol L^–1^ PBS of pH 7.4 recorded at the 3D-printed composite electrodes: (red) TPU/CNT, (green) TPU/CNT/BDD (1:1), and (blue) TPU/CNT/BDD (1:2).

Finally, the treated 3D-printed composite electrodes were subjected to electrochemical detection of 1 mmol L^–1^ dopamine in PBS (10 mmol L^–1^, pH 7.4). Dopamine, chosen as a model organic compound, is frequently used in electrochemical studies^23,34,46^ due to its well-defined redox mechanism and its biological significance as a neurotransmitter in the human body. Cyclic voltammetry of dopamine was initially conducted at all three types of 3D-printed electrodes, and the results obtained are shown in Figure S5. These measurements revealed significant background currents, particularly in the case of TPU/CNT/BDD samples, which resulted in poorly shaped cyclic voltammograms that were difficult to evaluate reliably. Consequently, we decided to proceed with differential pulse voltammetry, a more sensitive voltammetric technique that effectively suppresses background currents and facilitates the development of more distinct peaks. Differential pulse voltammograms, depicted in Figure 6, reveal varied responses among the tested composite electrodes. Specifically, the TPU/CNT electrode showed negligible signals, evidenced only by a slight current increase of 0.3 μA from the blank voltammogram, which could be ascribed to dopamine oxidation. However, the integration of BDD into the polymer matrix markedly enhanced dopamine detection, as indicated by the appearance of a well-defined peak ascribed to dopamine oxidation reaction, on both BDD-containing composite electrodes. In particular, the TPU/CNT/BDD (1:1) electrode exhibited an oxidation peak at +0.173 V with a peak height of 10.9 μA, whereas the TPU/CNT/BDD (1:2) electrode recorded a peak potential of +0.142 V and a peak height of 7.5 μA. Clearly, these first results highlight the beneficial impact of BDD incorporation on the electrochemical performance of the 3D-printed flexible composite electrodes, successfully validating the proof-of-concept. However, it is important to note that for dopamine, a more complex organic compound, the peak current did not increase proportionally with the increased BDD content, as was observed with the simple inorganic redox markers. This indicates that the filament (electrode) composition needs to be carefully optimized for the specific analyte of interest to maximize performance of the 3D-printed composite electrode.

## 4 Conclusion

We successfully pioneered the fabrication of 3D-printed flexible electrodes comprising CNT and BDD microparticles and manifested notable conductivity, electrochemical performance, desired structural integrity, and flexibility. The multi-faceted filament fabrication process was first addressed, which was essential for assuring homogeneous filament formulations and their consequent optimized printability. This led to effective 3D printing, based on fused deposition modeling, of highly flexible diamond-containing electrodes. Importantly, the herein developed fabrication route is considerably faster, more feasible, and significantly cheaper compared to previously reported methods, as it eliminates the need for a CVD reactor for thin-film diamond growth, inaccessible to many research groups, and costly cleanroom processing. The electrical conductivity of the filaments increased with higher BDD content, rising from 0.12 S m^–1^ for TPU/CNT to an order of magnitude higher value of 1.2 S m^–1^ for TPU/CNT/BDD (1:2). Notably, the electroactivity of the as-printed electrodes was significantly enhanced through a surface treatment procedure, primarily involving DMF-assisted partial dissolution of the insulating TPU polymer with an optimal incubation time of 20 min. This treatment not only improved the conductivity but also distinctly highlighted the compositional differences in the 3D-printed flexible electrodes, as thoroughly monitored through CV experiments with established redox markers. Particularly, the Δ*E*_p_ was reduced by 38% and *k*^0^_app_ increased by 2.1 times for [Ru(NH_3_)_6_]^3+/2+^ at the TPU/CNT/BDD (1:2) electrode, compared to the TPU/CNT electrode without BDD fillers. Similarly, for [Fe(CN)_6_]^3–/4–^, the lowest Δ*E*_p_ of 122 mV and the highest *k*^0^_app_ of 1.1 × 10^–3^ cm s^–1^ were achieved with the electrode containing the highest amount of fillers, i.e. TPU/CNT/BDD (1:2). The functionality of the flexible TPU/CNT/BDD composite electrodes was validated by the electrochemical detection of dopamine, as they exhibited well-developed oxidation signals with peak heights ranging from 7.5 to 10.9 μA, whereas the TPU/CNT composite electrodes showed only a negligible signal. Additionally, this study is the first to report the use of TPU as the primary polymer for filament production and the 3D printing of electrochemical sensors.

All in all, the synergy of diamond particles and carbon nanotubes within a flexible polymer matrix, coupled with 3D printing technology, holds the potential to revolutionize the fabrication of flexible diamond-containing devices with tailored shapes and sizes. This successful demonstration can boost research efforts targeting the development of innovative, flexible devices based on diamond, expanding their use in areas where flexibility is crucial, including neuroscience, biomedicine, health monitoring, and food safety.

## Supporting Information Available

Materials and chemicals used; additional experimental details on the preparation of composite pellets, filament extrusion, 3D printing process, and electrical characterization; BDD powder analysis; graphs (PDF)
Video demonstrating the flexibility of TPU/CNT/BDD electrode (MP4)
