# Supplementary material for: Advanced 3D-Printed Flexible Composite Electrodes of Diamond, Carbon Nanotubes, and Thermoplastic Polyurethane
Source: ACS Appl Polym Mater. 2024 Nov 19;6(23):14638–47. doi: 10.1021/acsapm.4c02748 (PMC11651389; doi:10.1021/acsapm.4c02748)
Supplement: Supplementary file 1 — ap4c02748_si_001.pdf [file ap4c02748_si_001.pdf]

Supporting Information

Advanced 3D-Printed Flexible Composite Electrodes of Diamond, Carbon Nanotubes, and Thermoplastic Polyurethane

Simona Baluchová[a,b]*, Stach van Leeuwen[a], Baris Kumru[c], Josephus G. Buijnsters[a,]*

[a] *Department of Precision and Microsystems Engineering, Faculty of Mechanical Engineering, Delft University of Technology, Mekelweg 2, 2628 CD Delft, the Netherlands*

[b] *Department of Analytical Chemistry, Faculty of Science, Charles University, Albertov 6, 128 00 Prague, Czech Republic*

[c] *Department of Aerospace Structures and Materials, Faculty of Aerospace Engineering, Delft University of Technology, Kluyverweg 1, 2629 HS Delft, the Netherlands*

Corresponding authors: simona.baluchova@natur.cuni.cz; j.g.buijnsters@tudelft.nl

A. Experimental

A1. Chemicals and materials

Thermoplastic polyurethane (TPU) pellets (~5 mm in length) were supplied by 123-3D.nl, the Netherlands. Boron-doped diamond (BDD) powder of high-pressure high-temperature origin (particle size of 0.1 – 1 µm, doping level of 710 ppm) was sourced from UHD Ultrahard Tools Co., China. Multi-walled carbon nanotubes (CNTs) with a diameter of 9.5 nm, a length of 1.5 µm, and a volume resistivity of 10^{-4} Ω cm were acquired from Nanocyl SA., Belgium. In addition, the manufacturer declared a percolation threshold for electrical conductivity between 0.5 wt.% and 4.5 wt.% of CNTs in thermoplastics. A range of chemicals (with purity indicated in %) including sulfuric acid (96.0 %), potassium nitrite (≥ 99.0 %), hexaammineruthenium(III) chloride (≥ 98.0 %), potassium hexacyanoferrate(III) (≥ 99.0 %), phosphate buffered saline (tablets, 10 mM, pH 7.4), dopamine hydrochloride (≥ 98.0 %), dimethylformamide (DMF, 99.5 %), acetone, and isopropanol was purchased from Sigma Aldrich, the Netherlands. All materials and chemicals were used as-received. All aqueous solutions were prepared using deionized water (resistivity of >18.0 MΩ cm), purified with a LWTN Genie A system (Laboratorium Water Technologie Nederland).

A2. Preparation of composite pellets

The fabrication of TPU/CNT/BDD composite pellets was conducted as follows: Initially, TPU pellets were dissolved in DMF at a 1:10 (g mL^{-1}) ratio, magnetically stirred for 2 h at 700 rpm, and maintained at 60 °C until complete dissolution. CNTs were then gradually introduced into the solution and stirred magnetically for another 2 h at 500 rpm and 60 °C. This was followed by ultrasonication of the composite dispersion for 1 h using an Emag Emmi-60 HC, operating at full power. BDD micropowder was subsequently added, stirred for 2 h, and ultrasonicated (1 h). The composite dispersion was left stirring overnight at 500 rpm at room temperature to ensure good homogeneity. The selected duration times for stirring and sonication were chosen, and probably slightly over-estimated, to ensure a truly homogeneous dispersion of the fillers within the polymer matrix. This conservative approach also aimed at minimizing the risk of damaging the CNTs, particularly preventing them from breaking into short fragments, which occurs when more time-efficient techniques are employed, such as high-power sonication. Such damage could potentially compromise the ability of CNTs to form an effective conducting network in the 3D-printed composite electrodes. The mixture was then introduced in deionized water to form composite precipitates, which were dried in an oven at 165 °C for 2 h to remove residual DMF and water. Finally, the dried composites were manually cut into pellets (10 – 20 mm in size) suitable for extrusion. Composite pellets containing only TPU/CNTs or TPU/BDD were prepared using the same procedure, excluding BDD powder or CNTs, respectively.

A3. Filament extrusion

The extrusion process utilized a Felfil Evo single-screw extruding instrument (Felfil, Italy), equipped with adjustable extrusion temperature and controlled extrusion speed or flow rate. To regulate the filament diameter, the hot extruded filament was passed over cooling fans and then to a spooling device featuring a stepping motor to maintain a consistent speed and diameter. Manual intervention was occasionally employed to pull the filament from the extruder for enhanced diameter control. The extrusion temperature was maintained between 195 – 215 °C, with an extrusion speed of 3-5 rpm, and a puller speed of 0.4 – 0.6 m min^{-1}. The extruded filament was manually cut into pieces with 10 – 20 mm lengths and re-extruded. This process was repeated until a dense, non-porous filament was achieved (see Figure S2(A) and Figure 2). The diameter of the filaments used for 3D-printing varied between 1.55 and 1.70 mm.

A4. 3D printing

SolidWorks 2022-2023 (Dassault Systèmes SE, France) facilitated the CAD design of the electrodes, with the CAD files subsequently exported as .STL format and converted to G-code using the open-source PrusaSlicer 2.5.0 (Prusa Research, Czech Republic). The 3D-printed electrodes were produced using a low-cost modified Prusa i3 MK3S+ printer based on fused deposition modelling (FDM; Prusa Research, Czech Republic). Modifications included replacing the original hot-end with one capable of withstanding higher temperatures between 250 °C and 300 °C, and employing a 0.8 mm ruby nozzle to prevent blockage from potential diamond aggregates and resist diamond abrasiveness. Key printing parameters for FDM-based composites are summarized in Table S1. The free-standing electrodes, measuring 25×25×1 mm^3 (width×length×thickness), had an average print time of 3 min per sample.

Table S1 Printing parameters used in the FDM fabrication procedure.

Print speed ($mm\ s^{-1}$)	Layer thickness (mm)	T_{nozzle} (°C)	T_{bed} (°C)	Raster angle (°)	Fill density (%)
20	0.3	215 - 250	50	45	100

A5. Electrical characterization

The electrical properties of the 3D-printed composites were evaluated using a Voltcraft CV820-1 digital multimeter, equipped with two probes. These probes were manually positioned at fixed distances on the composite filaments to measure resistance, which was then converted to volume resistivity (ρ) using the equation (Eq. S1):

$$\rho = R \times (A/L) \qquad \text{(Eq. S1)}$$

where R represents resistance, A the surface area, and L the distance between the probes or length of the conductive path. Subsequently, conductivity (σ) was calculated as the inverse of resistivity (Eq. S2):

$$\sigma = 1 / \rho \qquad \text{(Eq. S2)}$$

B. Figures & Results

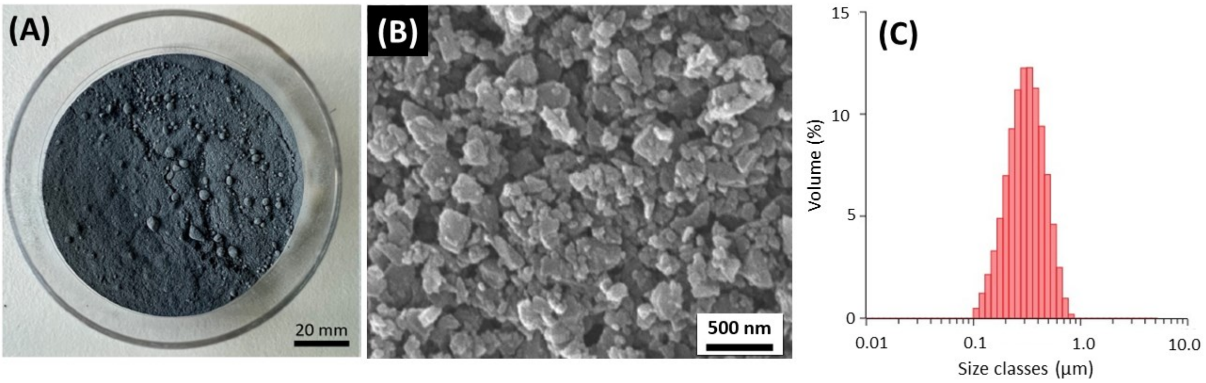

Figure S1. BDD powder used in this study: (A) photograph, (B) scanning electron micrograph, and (C) particle size distribution.

Prior to filament fabrication, commercially acquired BDD powder was subjected to scanning electron microscopic and particle size analysis. A photograph of the BDD powder, shown in Figure S1(A), reveals its dark grey color due to boron doping. The scanning electron micrograph of the powder, depicted in Figure S1(B), indicates that the majority of the particles possess a blocky and irregular shape, and are sub-micron in size. As illustrated in Figure S1(C), results of laser diffraction analysis confirmed that the BDD particle sizes range from 0.1 to 1.0 μm, with the most prevalent sizes being approximately 0.2 – 0.4 μm.

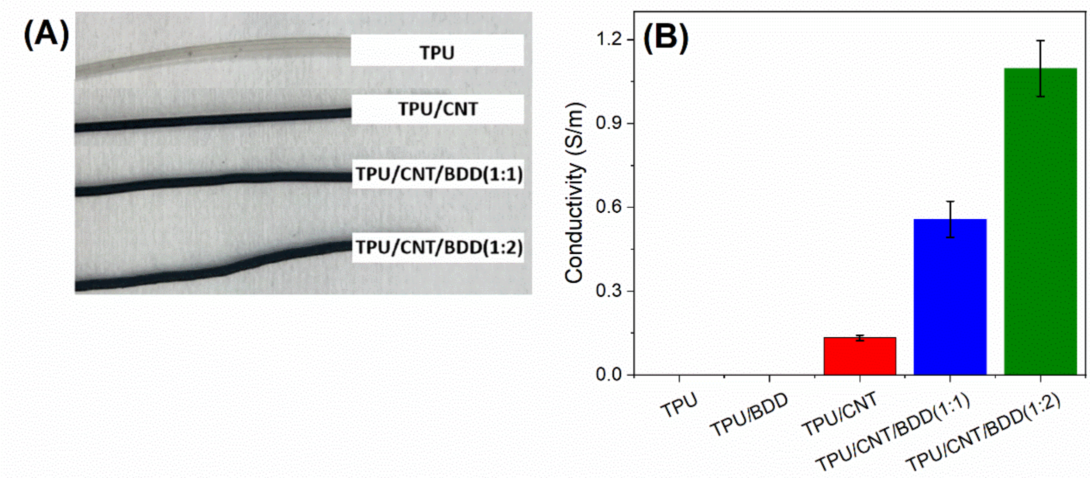

Figure S2. (A) A photograph of the various extruded filaments and (B) their measured electrical conductivity (evaluated for $n = 10$).

The volume resistivity of the 3D-printed composites was determined using the procedure outlined in Section A5 using Eq. S1, with electrical conductivity subsequently calculated as the inverse of resistivity (Eq. S2). The electrical conductivity of herein fabricated filaments is graphically displayed in Figure S2(B). The measured resistivity of the filament containing only TPU was below the detectable threshold and confirmed the insulating property of the polymer. However, upon incorporation of CNTs (10 wt.%) into the TPU/CNT composite, a notable increase in a conductivity of 0.12 S m^{-1} (corresponding to a volume resistivity of 757 Ω cm) was immediately observed. This increase indicates that the CNT concentration significantly surpasses the percolation threshold of 0.5 - 4.5 wt.% (as established by Nanocyl). After introducing BDD particles in the filament composite, the volume resistivity gradually dropped with increasing BDD concentration and a value of 92 Ω cm was recorded for the TPU/CNT/BDD (1:2) filament. This corresponds to a conductivity of 1.2 S m^{-1}, which is one order of magnitude higher than the value measured for the TPU/CNT filament.

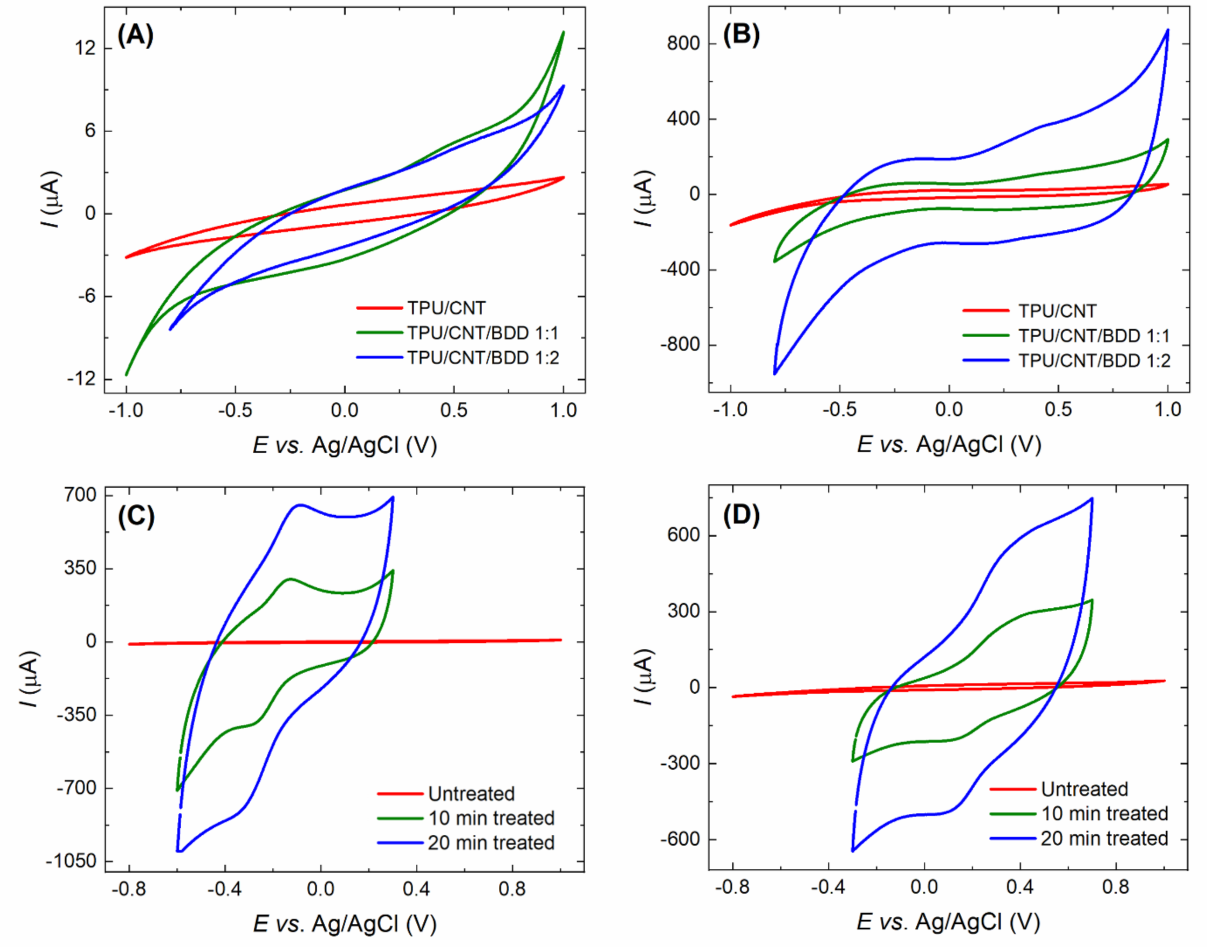

Figure S3. (A, B) Cyclic voltammograms recorded at a scan rate of 100 mV s^{-1} in a 0.5 mol L^{-1} KNO_3 solution on (A) as-printed and (B) treated composite electrodes. (C, D) Cyclic voltammograms recorded in the solutions of (C) $[Ru(NH_3)_6]^{3+/2+}$ and (D) $[Fe(CN)_6]^{3-/4-}$ (both 1 mmol L^{-1} in 0.5 mol L^{-1} KNO_3) at the TPU/CNT/BDD (1:2) electrode (v of 100 mV s^{-1}).

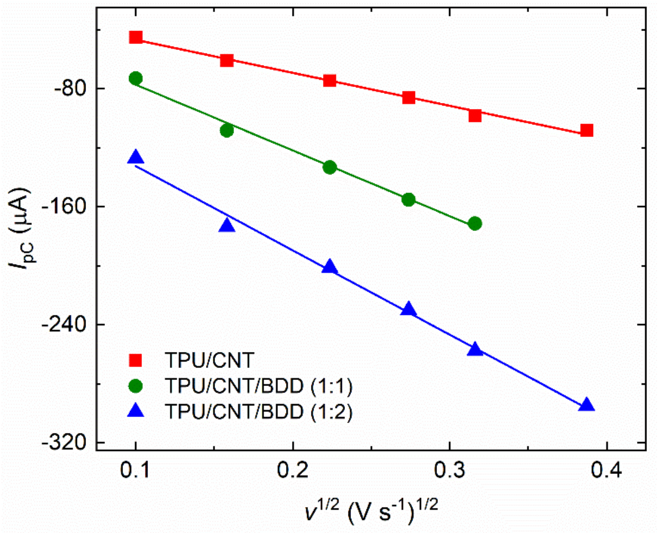

Figure S4. Linear dependencies of the cathodic peak currents (I_{pC}) on the square root of the scan rate ($v^{1/2}$) evaluated for the treated 3D-printed composite electrodes. These dependencies were plotted based on CV measurements conducted using 1 mmol L^{-1} $[Ru(NH_3)_6]^{3+/2+}$ in 0.5 mol L^{-1} KNO_3.

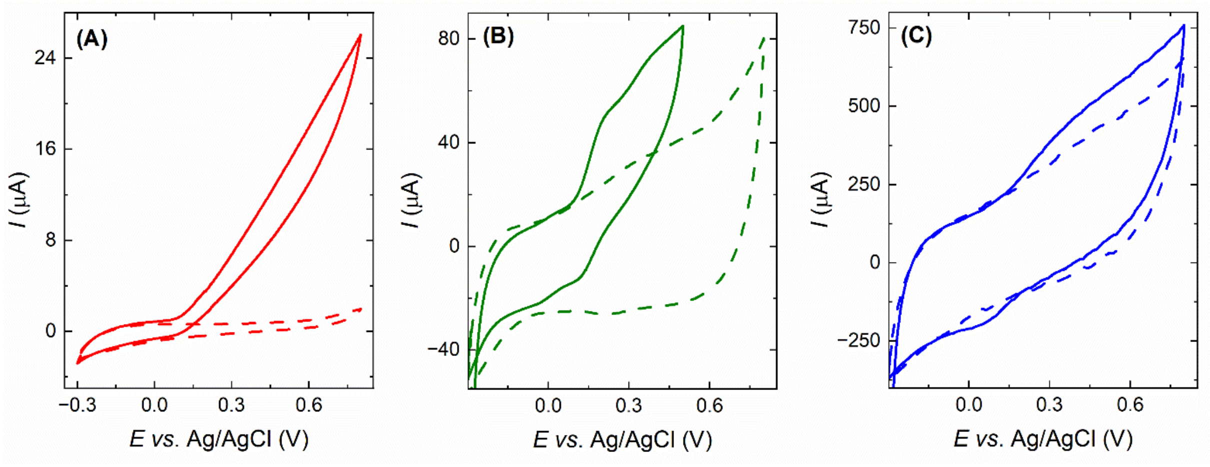

Figure S5. Cyclic voltammograms of 1 mmol L^{-1} dopamine in 10 mmol L^{-1} PBS of pH 7.4 recorded at the treated 3D-printed composite electrodes: (A) TPU/CNT (scan rate 10 mV s^{-1}), (B) TPU/CNT/BDD (1:1; scan rate 10 mV s^{-1}), and (C) TPU/CNT/BDD (1:2; scan rate 100 mV s^{-1}). Dashed lines represent supporting electrolyte.

Cyclic voltammetry of dopamine was initially conducted at all three types of 3D-printed electrodes and the results obtained are shown in Figure S5. These measurements revealed significant background currents, particularly in the case of TPU/CNT/BDD samples, which resulted in poorly shaped cyclic voltammograms that were difficult to evaluate reliably.
